# Characterization of tracheotomized patients after spontaneous subarachnoid hemorrhage

**DOI:** 10.1097/MD.0000000000021057

**Published:** 2020-07-10

**Authors:** Yu-Ming Chang, Tsung-Han Lee, Chen-Chieh Liao, Yu-Hua Huang

**Affiliations:** a Department of Neurosurgery, Kaohsiung Chang Gung Memorial Hospital and Chang Gung University College of Medicine; b Graduate Institute of Medicine, College of Medicine, Kaohsiung Medical University, Kaohsiung; c Department of Neurosurgery, Pingtung Christian Hospital, Pingtung, Taiwan; d Department of Neurosurgery, Xiamen Chang Gung Hospital, Xiamen, Fujian, China.

**Keywords:** complication, risk factor, subarachnoid hemorrhage, tracheostomy

## Abstract

Spontaneous subarachnoid hemorrhage (SAH) is a catastrophic event with high disability and fatality rates. Post-SAH survivors may require prolonged intubation with the assistance of mechanical ventilators, and some patients will undergo tracheostomy to facilitate their pulmonary hygiene and airway protection. The aim of this study is to identify the incidence and risk factors of the need for tracheostomy after spontaneous SAH. We used a retrospective approach and enrolled 838 adult patients with a primary diagnosis of spontaneous SAH who survived >7 days after hospitalization. Medical information was retrieved from the administrative database utilizing diagnostic and procedure codes by the International Classification of Diseases, Ninth Revision, Clinical Modification. Patients with first-ever SAH included 329 men and 509 women, and their average age was 56.9 ± 14.4 years, ranging between 18 and 91 years. Fifty-eight of these 838 patients underwent tracheostomy procedures, and the overall incidence was 6.9%. In a multivariate logistic regression model, the independent risk factors of the need for tracheostomy were underlying diabetes mellitus (*P* = .02), hydrocephalus (*P* < .01), and pneumonia (*P* < .01). The mean duration of hospital stay was 26.0 ± 15.3 and 16.8 ± 12.2 days for patients with and without a tracheostomy, respectively (*P* < .01). In conclusion, a significant percentage of post-SAH survivors underwent tracheostomy during acute hospitalization. Attention to independent risk factors, including preexisting diabetes mellitus, concomitant hydrocephalus, and nosocomial pneumonia, is essential for timely patient selection for tracheostomy.

## 1 Introduction

Spontaneous subarachnoid hemorrhage (SAH) is a devastating cerebrovascular event, and the acute mortality rate ranges between 20% and 40%.^[1,2]^ Even though patients survive from the primary impact of intracranial hemorrhage, they usually have a remarkable probability for medical complications that are highly associated with unfavorable outcomes of SAH.^[3,4]^ Moreover, the incidence of SAH increases with age, which has risen from 52.9 to 56.6 years over the past decades,^[5]^ and the elderly are theoretically susceptible to medical morbidities. In addition, after SAH, risks exist for patients with a prognosis of severe disability or persistent vegetative state. Therefore, it raises the concern that post-SAH survivors often require prolonged intubation with the assistance of mechanical ventilators in the intensive care unit. These patients may have respiratory sequelae resulting from the disability of the pharyngeal protection reflex, persistence of excessive secretions, or inadequacy of spontaneous ventilation. Ultimately, a proportion of patients undergo tracheostomy to facilitate their pulmonary hygiene and airway protection.

The predictive ability to recognize who will need a permanent airway is important for earlier initiation of weaning from mechanical ventilation under controlled circumstances. It potentially prevents premature extubation leading to rapid respiratory failure and violent reintubation as an emergency and reduces the rate of secondary cerebral injury related to underestimated hypoxia.

As a result, the aim of this study was to determine the incidence of tracheostomy after spontaneous SAH and to assess risk factors that would identify the need for tracheostomy based on early clinical characteristics.

## 2 Materials and methods

This research was conducted at Kaohsiung Chang Gung Memorial Hospital, a tertiary referral medical center in Taiwan, and had been approved by the institutional review board. We retrieved medical information retrospectively from the administrative database, including the following clinical parameters: dates of admission and discharge; gender; marital status; age; diagnostic codes of the International Classification of Diseases, Ninth Revision, Clinical Modification (ICD-9-CM); procedure codes of ICD-9-CM; status at discharge; and related data. From January 2000 to December 2010, we identified 1094 hospital admissions with a primary diagnosis of SAH (ICD-9-CM code 430) and excluded 209 patients, who died within 7 days after SAH, and 47 patients who were younger than 18 years old, readmissions, or had incomplete documents. Finally, we enrolled 838 adult patients with SAH surviving from initial insult for further analysis.

The baseline characteristics were studied, and included demographics and underlying diseases of hypertension (ICD-9-CM Codes 4010–4059), diabetes mellitus (ICD-9-CM Codes 2500–2509), hyperlipidemia (ICD-9-CM Codes 2720–2724), chronic pulmonary disease (ICD-9-CM Codes 490–505), chronic kidney disease (ICD-9-CM Codes 585–586), coronary artery disease (ICD-9-Codes 4140–4149), heart failure (ICD-9-CM Codes 4280–4289), liver disease (ICD-9-CM Codes 570–573), peptic ulcer disease (ICD-9-CM Codes 53,100–53,491), coagulopathy (ICD-9-CM Codes 2860–2869), or thrombocytopenia (ICD-9-CM Codes 2870–2875).

Patients who underwent a tracheostomy procedure were recognized when coded as 311, 3121, or 3129. We investigated other major therapeutic management, including surgical treatments for cerebral aneurysms (Procedure Codes 3951–3952), endovascular interventions for cerebral aneurysms (Procedure Code 3979), or blood product transfusion (Procedure Codes 9903–9907).

The patients suffered from medical complications, including diabetes insipidus (ICD-9-CM Code 2535), hypernatremia or hyperosmolarity (ICD-9-CM Code 2760), hyponatremia or hyposmolarity (ICD-9-CM Code 2761), hyperpotassemia (ICD-9-CM Code 2767), hypopotassemia (ICD-9-CM Code 2768), anemia (ICD-9-CM Codes 2851 & 2859), acute kidney failure (ICD-9-CM Codes 5845–5849), gastrointestinal hemorrhage (ICD-9-CM Codes 5780–5789), or pneumonia (ICD-9-CM Codes 481–486). Neurological complications included central nervous system infection (ICD-9-CM Codes 3200–3249), hydrocephalus (ICD-9-CM Codes 3313–3314), cerebral ischemia or infarction (ICD-9-CM Codes 4330–4371), convulsion (ICD-9-CM Code 7803), or hemiplegia (ICD-9-CM Codes 3420–3429). The length of hospital stay and condition at discharge were both recorded.

We evaluated the data using SPSS software (IBM SPSS Statistics, Version 20.0. Armonk, NY: IBM Corp.). Parameters were shown as numbers (percentage) or as a mean ± standard deviation. Intergroup differences were analyzed using the Student *t* test for continuous variables and the Chi-squared test or Fisher exact test for categorical variables. All parameters with a *P*-value < .05 were entered into a multivariable logistic regression to adjust for independent risk factors of tracheostomy after SAH. Statistical significance was defined as a *P* < .05.

## 3 Results

**Figure 1:**
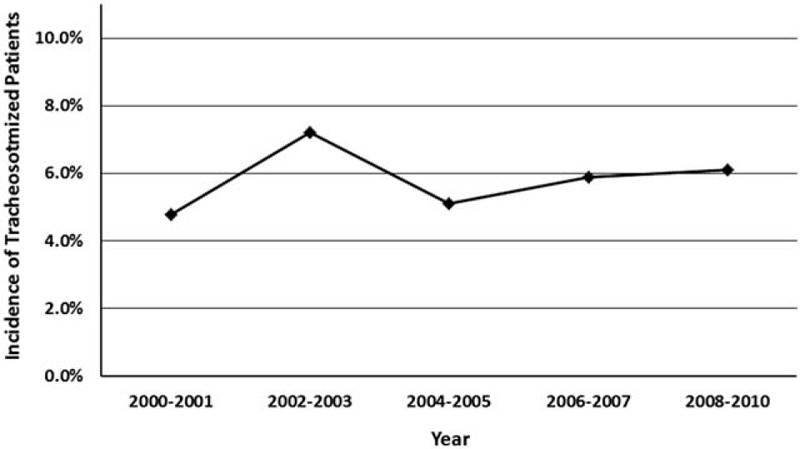
Incidence of tracheostomized patients after spontaneous subarachnoid hemorrhage over time.

The 838 patients with first-ever spontaneous SAH included 329 men and 509 women. The mean age at the time of diagnosis was 56.9 ± 14.4 years, ranging between 18 and 91 years old. The mean length of hospital stay was 17.4 ± 12.6 days. Fifty-eight of the 838 patients underwent tracheostomy procedures, and the overall incidence was 6.9%. During 2000 and 2010, the annual rate of tracheotomized patients did not change markedly and remained between 4% and 8% (Fig. 1).

Patients had the following underlying diseases: hypertension (377, 45.0%), diabetes mellitus (75, 8.9%), hyperlipidemia (13, 1.6%), chronic pulmonary disease (13, 1.6%), chronic kidney disease (8, 1.0%), coronary artery disease (7, 0.8%), heart failure (7, 0.8%), liver disease (26, 3.1%), peptic ulcer (14, 1.7%), coagulopathy (4, 0.5%), and thrombocytopenia (9, 1.1%). Of these 838 patients, 339 (40.5%) underwent surgical treatments and 164 (19.6%) underwent endovascular interventions for cerebral aneurysms. In addition, 339 (40.5%) cases received blood product transfusions.

The in-hospital morbidities were as follows: diabetes insipidus (7, 0.8%), hypernatremia or hyperosmolarity (12, 1.4%), hyponatremia or hyposmolarity (40, 4.8%), hyperpotassemia (6, 0.7%), hypopotassemia (70, 8.4%), anemia (92, 11.0%), acute kidney failure (9, 1.1%), gastrointestinal hemorrhage (26, 3.1%), pneumonia (102, 12.2%), central nervous system infection (38, 4.5%), hydrocephalus (295, 35.2%), cerebral ischemia or infarction (90, 10.7%), convulsion (48, 5.7%), and hemiplegia (56, 6.7%).

In a comparison of clinical features of patients with or without a tracheostomy, statistical analysis identified the following parameters with a *P*-value < .05: age (*P* = .03), diabetes mellitus (*P* = .01), pneumonia (*P* < .01), central nervous system infection (*P* < .01), and hydrocephalus (*P* < .01) (Table 1). All these factors were entered into multivariable regression analysis, and the independent risk factors of the need for tracheostomy following SAH included underlying diabetes mellitus (*P* = .02), pneumonia (*P* < .01), and hydrocephalus (*P* < .01) (Table 2).

**Table 1:**
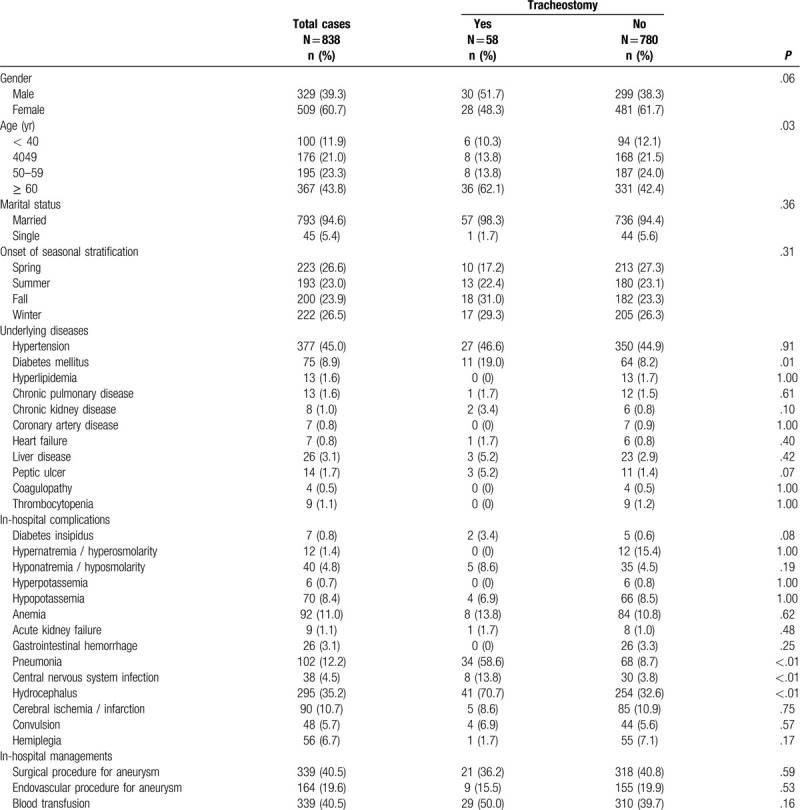
Comparisons of clinical features between patients with or without tracheostomy after subarachnoid hemorrhage.

**Table 2:**
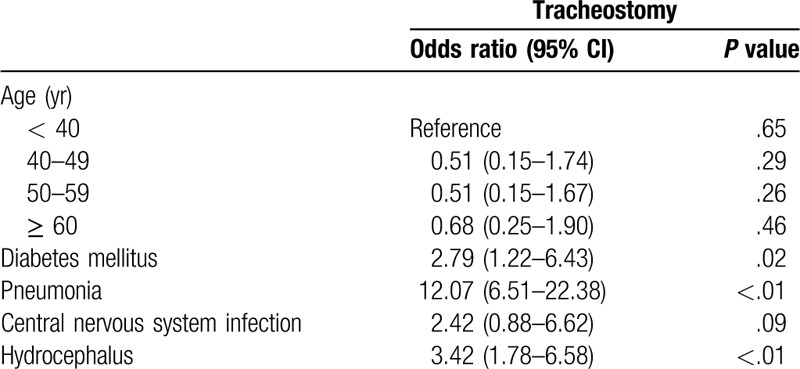
Multivariable analysis of independent risk factors for tracheostomy after subarachnoid hemorrhage.

The mean duration of hospital stay was 26.0 ± 15.3 and 16.8 ± 12.2 days for patients with and without tracheostomy, respectively (*P* < .01). At discharge, 6 of the 58 patients with tracheostomy (10.3%), and 96 of the 780 patients without tracheostomy (12.3%) had died after the first 7 days; no remarkable difference in the late mortality rate was observed between the 2 study groups (*P* = .82).

## 4 Discussion

As a neurological emergency with serious prognostic impact, SAH is an intensive topic of clinical research, and the management of medical complications are extensively investigated.^[3,4,6]^ This investigation is one of the largest Asian cohort studies, and the results showed up to 6.9% of the 838 adults with SAH underwent tracheostomy during acute hospitalization. In an investigation focusing on respiratory issues of acute stroke, Lahiri et al reported that 38.5% of post-SAH patients developed acute respiratory failure necessitating mechanical ventilation, and 16.6% of these patients who received mechanical ventilation underwent tracheostomy.^[7]^ It is estimated that 6.4% of their SAH victims had a tracheostomy, which was similar to our observation. In comparison, the need for tracheostomy was 4.6% after intracerebral hemorrhage and only 1.3% in patients with ischemic stroke.^[7]^ Therefore, though SAH accounts for only 5% to 10% of all strokes,^[6,8]^ it is accompanied by the highest percentage of resultant respiratory sequelae, which deserves more attention by neurointensivists.

Patients with poor-grade SAH are usually intubated at the resuscitation or intervention site, and a secure airway is maintained for subsequent critical monitoring. Bösel described 2 main scenarios in which tracheostomy is considered after acute stroke.^[9]^ The first is to bridge swallowing therapy and prevent aspiration, and the second is as part of weaning from the ventilator if extubation fails or is deemed not feasible. Our prior study disclosed that SAH survivors had a remarkable incidence of resultant deficits,^[10]^ and tracheostomy theoretically played an integral role for such patients with restricted recovery. However, potential procedure-related complications, such as pneumothorax, tracheal stenosis, or surgical site infection, cannot be ignored,^[11,12]^ so clinical judgment regarding converting to tracheostomy should be made with both caution and more experienced guidance.

Several studies have defined the stroke population that will require intubation and eventual tracheostomy. In an earlier pilot clinical trial, stroke-related early tracheostomy versus prolonged intubation was done to estimate tracheostomy necessity by combining 3 parameter groups, including neurological functions, neurological lesions, and extracerebral organ functions or procedures.^[9,13]^ In addition, Steidl et al found that tracheostomized patients were primarily more hemorrhagic, more obese, had a suspicion of compromised protective reflexes, and often needed neurosurgical intervention.^[14]^ Interestingly, SAH itself is a factor significantly associated with tracheostomy among hemorrhagic strokes.^[12]^ In our study, we ultimately identified diabetes mellitus, pneumonia, and hydrocephalus as independent variables to predict the need for post-SAH tracheostomy, and specifically characterized this more complicated and aggressive subtype of stroke.

The meta-analysis provided evidence that hyperglycemia at admission increases the risk of poor outcomes after SAH.^[15]^ In our experimental study, hyperglycemia-preconditioned rats had worse neurological functions following SAH than those with either hyperglycemia or SAH alone, which reinforced clinical findings.^[16]^ It is reasonable to hypothesize that factors affecting the prognosis of patients following SAH also may be responsible for an increased risk of tracheotomy, and the connection between diabetes mellitus and tracheostomy shown in this study seems not surprising. The acute phase of stroke is usually accompanied by a humoral surge in cortisol and catecholamines,^[17]^ which results in elevated blood glucose levels regardless of a previous diabetes diagnosis. It remains controversial whether outcomes improve with strict normalization of blood glucose following SAH because the drawback of intensive glycemic control is an increased frequency of hypoglycemic episodes.^[18,19]^ Thus, even we recognize that diabetes mellitus is predictive of the need for tracheostomy, but an advanced investigation is required to clarify whether diabetic or glycemic treatment is helpful to reduce the occurrence of tracheostomy or respiratory complications.

Pneumonia is a common medical morbidity of SAH,^[3,4]^ and accounts for 12.2% of the cases in this study. At first, disability of the protection reflex and inadequacy of ventilation lead to impaired clearance of secretions and atelectasis. Subsequent pulmonary infection further limits the reserve capacity of the pulmonary system. As a result, respiratory failure may originate from post-SAH neurological dysfunction alone, or in combination with pneumonia. On the other hand, prolonged endotracheal intubation itself for impaired consciousness is risky for nosocomial pneumonia,^[20]^ which also affects ventilator weaning. Because pneumonia contributes substantively to respiratory failure or tracheostomy dependence after SAH, the incorporation of multiple interventions in bundles should be implemented in a timely manner to prevent the occurrence of pneumonia.

Acute hydrocephalus is a major complication following SAH, and several risk factors of hydrocephalus are well documented, such as the presence of intraventricular blood, high Fisher score, aneurysmal rebleeding, posterior circulation aneurysm location, high Hunt and Hess scale score, and older age.^[21,22]^ These factors are usually associated with initial clinical severity or subarachnoid hematoma volume. With a higher amount of blood cells and corresponding products, the basal cisterns and outflow tract of cerebrospinal fluid are blocked, and thus, the presence of hydrocephalus can be viewed as a sign of severe SAH. Because of the higher probability of a reduced conscious level and delayed functional recovery, this might explain why hydrocephalus is significantly relevant to the need for tracheostomy after SAH.

There were important limitations to our study. First, the utility of ICD-9-CM coding for the clinical investigation was occasionally debated, because the details, such as Hunt and Hess grading, Fisher grading, features of cerebral aneurysms, or serial functional status, was difficult to be accessed. Second, the etiology of intubation or mechanical ventilation was unknown, thereby limiting the understanding of the association between the parameters of interest. Third, since this analysis lacked long-term outcomes, the actual effectiveness of tracheostomy among survivors was unclear. Even with these issues of the study, we consider the data useful as reliable information for clinicians and families, and crucial from a public health perspective.

## 5 Conclusion

The incidence of the need for tracheostomy was 6.9% in survivors after spontaneous SAH. Attention to independent risk factors, including preexisting diabetes mellitus, concomitant hydrocephalus, and nosocomial pneumonia, is essential for timely patient selection for tracheostomy.

## Author contributions

**Conceptualization:** Yu-Ming Chang.

**Investigation:** Yu-Ming Chang, Tsung-Han Lee.

**Validation:** Tsung-Han Lee.

**Reviewing, editing:** Chen-Chieh Liao.

**Supervision:** Yu-Hua Huang.

## References

[1] Feigin VL Lawes CM Bennett DA Worldwide stroke incidence and early case fatality reported in 56 population-based studies: a systematic review. Lancet Neurol 2009;8:355–69. 19233729 10.1016/S1474-4422(09)70025-0

[2] Gonzalez-Perez A Gaist D Wallander MA Mortality after hemorrhagic stroke: data from general practice (The Health Improvement Network). Neurology 2013;81:559–65. 23843467 10.1212/WNL.0b013e31829e6eff

[3] Wartenberg KE Schmidt JM Claassen J Impact of medical complications on outcome after subarachnoid hemorrhage. Crit Care Med 2006;34:617–23. 16521258 10.1097/01.ccm.0000201903.46435.35

[4] Wartenberg KE Mayer SA Medical complications after subarachnoid hemorrhage. Neurosurg Clin N Am 2010;21:325–38. 20380973 10.1016/j.nec.2009.10.012

[5] Rincon F Rossenwasser RH Dumont A The epidemiology of admissions of nontraumatic subarachnoid hemorrhage in the United States. Neurosurgery 2013;73:217–22. 23615089 10.1227/01.neu.0000430290.93304.33

[6] Suarez JI Tarr RW Selman WR Aneurysmal subarachnoid hemorrhage. N Engl J Med 2006;354:387–96. 16436770 10.1056/NEJMra052732

[7] Lahiri S Mayer SA Fink ME Mechanical ventilation for acute stroke: a multi-state population-based study. Neurocrit Care 2015;23:28–32. 25487123 10.1007/s12028-014-0082-9

[8] de Rooij NK Linn FH van der Plas JA Incidence of subarachnoid haemorrhage: a systematic review with emphasis on region, age, gender and time trends. J Neurol Neurosurg Psychiatry 2007;78:1365–72. 17470467 10.1136/jnnp.2007.117655 PMC2095631

[9] Bosel J Use and timing of tracheostomy after severe stroke. Stroke 2017;48:2638–43. 28733479 10.1161/STROKEAHA.117.017794

[10] Huang YH Liao CC Yang KY Demographics and short-term outcomes of spontaneous subarachnoid hemorrhage in young adults. World Neurosurg 2017;102:414–9. 28347893 10.1016/j.wneu.2017.03.069

[11] Dasenbrock HH Rudy RF Gormley WB The timing of tracheostomy and outcomes after aneurysmal subarachnoid hemorrhage: a nationwide inpatient sample analysis. Neurocrit Care 2018;29:326–35. 30298335 10.1007/s12028-018-0619-4

[12] McCann MR Hatton KW Vsevolozhskaya OA Earlier tracheostomy and percutaneous endoscopic gastrostomy in patients with hemorrhagic stroke: associated factors and effects on hospitalization. J Neurosurg 2019;132:87–93. 30611136 10.3171/2018.7.JNS181345

[13] Bosel J Schiller P Hook Y Stroke-related early tracheostomy versus prolonged orotracheal intubation in neurocritical care trial (SETPOINT): a randomized pilot trial. Stroke 2013;44:21–8. 23204058 10.1161/STROKEAHA.112.669895

[14] Steidl C Bosel J Suntrup-Krueger S Tracheostomy, extubation, reintubation: airway management decisions in intubated stroke patients. Cerebrovasc Dis 2017;44:1–9. 10.1159/000471892 28395275

[15] Kruyt ND Biessels GJ de Haan RJ Hyperglycemia and clinical outcome in aneurysmal subarachnoid hemorrhage: a meta-analysis. Stroke 2009;40:e424–30. 19390078 10.1161/STROKEAHA.108.529974

[16] Huang YH Chung CL Tsai HP Hyperglycemia aggravates cerebral vasospasm after subarachnoid hemorrhage in a rat model. Neurosurgery 2017;80:809–15. 28379540 10.1093/neuros/nyx016

[17] Feibel JH Hardy PM Campbell RG Prognostic value of the stress response following stroke. JAMA 1977;238:1374–6. 578192

[18] Bilotta F Spinelli A Giovannini F The effect of intensive insulin therapy on infection rate, vasospasm, neurologic outcome, and mortality in neurointensive care unit after intracranial aneurysm clipping in patients with acute subarachnoid hemorrhage: a randomized prospective pilot trial. J Neurosurg Anesthesiol 2007;19:156–60. 17592345 10.1097/ANA.0b013e3180338e69

[19] Latorre JG Chou SH Nogueira RG Effective glycemic control with aggressive hyperglycemia management is associated with improved outcome in aneurysmal subarachnoid hemorrhage. Stroke 2009;40:1644–52. 19286596 10.1161/STROKEAHA.108.535534 PMC2705778

[20] Fernandez JF Levine SM Restrepo MI Technologic advances in endotracheal tubes for prevention of ventilator-associated pneumonia. Chest 2012;142:231–8. 22796845 10.1378/chest.11-2420 PMC3418858

[21] Chen S Luo J Reis C Hydrocephalus after subarachnoid hemorrhage: pathophysiology, diagnosis, and treatment. BioMed Res Int 2017;2017:8584753. 28373987 10.1155/2017/8584753 PMC5360938

[22] Wilson CD Safavi-Abbasi S Sun H Meta-analysis and systematic review of risk factors for shunt dependency after aneurysmal subarachnoid hemorrhage. J Neurosurg 2017;126:586–95. 27035169 10.3171/2015.11.JNS152094

